# Statin Use and Risk of Lung Cancer: a Meta-Analysis of Observational Studies and Randomized Controlled Trials

**DOI:** 10.1371/journal.pone.0077950

**Published:** 2013-10-25

**Authors:** Jinliang Wang, Cheng Li, Haitao Tao, Yao Cheng, Lu Han, Xiaoyan Li, Yi Hu

**Affiliations:** Department of general oncology, Chinese PLA General Hospital, Beijing, People's Republic of China; Univesity of Texas Southwestern Medical Center at Dallas, United States of America

## Abstract

Clinical studies have shown that statin use may alter the risk of lung cancer. However, these studies yielded different results. To quantify the association between statin use and risk of lung cancer, we performed a detailed meta-analysis. A literature search was carried out using MEDLINE, EMBASE and COCHRANE database between January 1966 and November 2012. Before meta-analysis, between-study heterogeneity and publication bias were assessed using adequate statistical tests. Fixed-effect and random-effect models were used to calculate the pooled relative risks (RR) and corresponding 95% confidence intervals (CIs). Subgroup analyses, sensitivity analysis and cumulative meta-analysis were also performed. A total of 20 (five randomized controlled trials, eight cohorts, and seven case–control) studies contributed to the analysis. Pooled results indicated a non-significant decrease of total lung cancer risk among all statin users (RR = 0.89, 95% CI [0.78, 1.02]). Further, long-term statin use did not significantly decrease the risk of total lung cancer (RR = 0.80, 95% CI [0.39 , 1.64]). In our subgroup analyses, the results were not substantially affected by study design, participant ethnicity, or confounder adjustment. Furthermore, sensitivity analysis confirmed the stability of results. The findings of this meta-analysis suggested that there was no significant association between statin use and risk of lung cancer. More studies, especially randomized controlled trials and high quality cohort studies are warranted to confirm this association.

## Introduction

Lung cancer is the leading cause of cancer death worldwide[1,2]. The age-adjusted incidence rate of lung cancer was 62.6 per 100,000 men and women per year, and the age-adjusted death rate was 50.6 per 100,000 men and women per year[3]. 3-hydroxy-3-methylglutaryl-coenzyme A reductase inhibitors (statins) are the most commonly used drugs in the treatment of hypercholesterolemia, which potently reduce plasma cholesterol levels. Their efficacy on cardiovascular events has been proven irrefutably for both reduction of morbidity and mortality[4,5]. Rodent studies suggested that statins may be carcinogenic[6]. However, several preclinical studies have shown that statins may have potential anticancer effects through arresting of cell cycle progression[7], inducing apotosis[8,9], suppressing angiogenesis[10,11], and inhibiting tumor growth and metastasis[12,13]. For lung cancer, some experimental studies have found that statin may induces apoptosis[14–18], inhibit tumor growth[19–22], angiogenesis[23], as well as metastasis[24]. Further, statin may overcome drug resistance in human lung cancer[25]. Now there are some studies investigating the association between statin use and lung cancer, however, the existing results are controversial. To better understand this issue, we carried out a meta -analysis of existing randomized controlled trials (RCT) and observational studies that investigated the association between statins use and the risk of developing lung cancer.

## Materials and Methods

### Literature Search

The meta-analysis was undertaken in accordance with the Preferred Reporting Items for Systematic Reviews and Meta-Analyses (PRISMA)[26]. A literature search was carried out using MEDLINE, EMBASE and COCHRANE databases between January 1966 and November 2012. There were no restriction of origin and languages. Search terms included: ‘‘hydroxymethylglutaryl-CoA reductase inhibitor(s)’’ or ‘‘statin(s)’’ or ‘‘lipid-lowering agent(s)’’ and ‘‘cancer(s)’’ or ‘‘neoplasm(s)’’ or ‘‘malignancy(ies)’’. The reference list of each comparative study and previous reviews were manually examined to ﬁnd additional relevant studies.

### Study selection

Two reviewers independently selected eligible trials. Disagreement between the two reviewers was settled by discussing with the third reviewer. Inclusion criteria were: (i) an original study comparing statin treatment with an inactive control (placebo or no statins), (ii) adult study participants (18 years or older), (iii) presented odds ratio (OR), relative risk (RR), or hazard ratio (HR) estimates with its 95% confidence interval (CI), or provided data for their calculation., and (iv)follow-up over one year. Studies without lung cancer assessment and those describing statin treatment in cancer or transplant patients were excluded. When there were multiple publications from the same population, only data from the most recent report were included in the meta-analysis and remaining were excluded . Studies reporting different measures of RR like risk ratio, rate ratio, HR, and OR were included in the meta-analysis. In practice, these measures of effect yield a similar estimate of RR, since the absolute risk of lung cancer is low.

### Data extraction

The following data was collected by two reviewers independently using a purpose-designed form: name of first author, publishing time, country of the population studied, study design, study period, patient characteristics, statin type, the RR estimates and its 95 % CIs, confounding factors for matching or adjustments. 

### Methodological quality assessment

The quality of included randomized controlled trials (RCT) was assessed using the tool of “risk of bias” according to the Cochrane Handbook. Sequence generation, allocation concealment, blinding, incomplete data and selective reporting were assessed, and each of them was graded as “yes(+)”, “no(-)” or “unclear(?)”, which reflected low risk of bias, high risk of bias and uncertain risk of bias, respectively. We used Newcastle-Ottawa scale to assess the methodologic quality of cohort and case–control studies. The Newcastle-Ottawa Scale contains eight items that are categorized three categories: selection (four items, one star each), comparability (one item, up to two stars), and exposure/outcome (three items, one star each). A ‘‘star’’ presents a ‘‘high-quality’’ choice of individual study. Two reviewers who were blinded regarding the source institution, the journal, and the authors for each included publication independently assess the methodologic quality. Disagreement between the two reviewers was settled by discussing with the third reviewer.

### Data synthesis and analysis

Heterogeneity was assessed using the Cochran Q and I^2^ statistics. For the Q statistic, a P value<0.10 was considered statistically significant for heterogeneity; for the I^2^ statistic, heterogeneity was interpreted as absent (I^2^: 0%–25%), low (I^2^: 25.1%–50%), moderate (I^2^: 50.1%–75%), or high (I^2^: 75.1%–100%)[27]. The overall analysis including all eligible studies was performed first, and subgroup analyses were performed according to (i) study design (RCT, cohort and case–control studies), (ii) study location, and (iii)control for confounding factors ( n ≥ 8, n ≤ 7) , to examine the impact of these factors on the association. We also assessed the link between long-term statin use and lung cancer risk. Pooled RR estimates and corresponding 95 % CIs were calculated using the inverse variance method. In the absence of a statistically signiﬁcant heterogeneity (I^2^: 0%–25%), fixed model was used; otherwise, random model was performed. To test the robustness of association and characterize possible sources of statistical heterogeneity, sensitivity analysis was carried out by excluding studies one-by-one and analyzing the homogeneity and effect size for all of rest studies. Publication bias was assessed using Begg and Mazumdar adjusted rank correlation test and the Egger regression asymmetry test[28,29]. All analyses were performed using Stata version 11.0 (StataCorp, College Station, TX).

## Results

### Search results and characteristics of studies included in the meta-analysis

**Figure 1:**
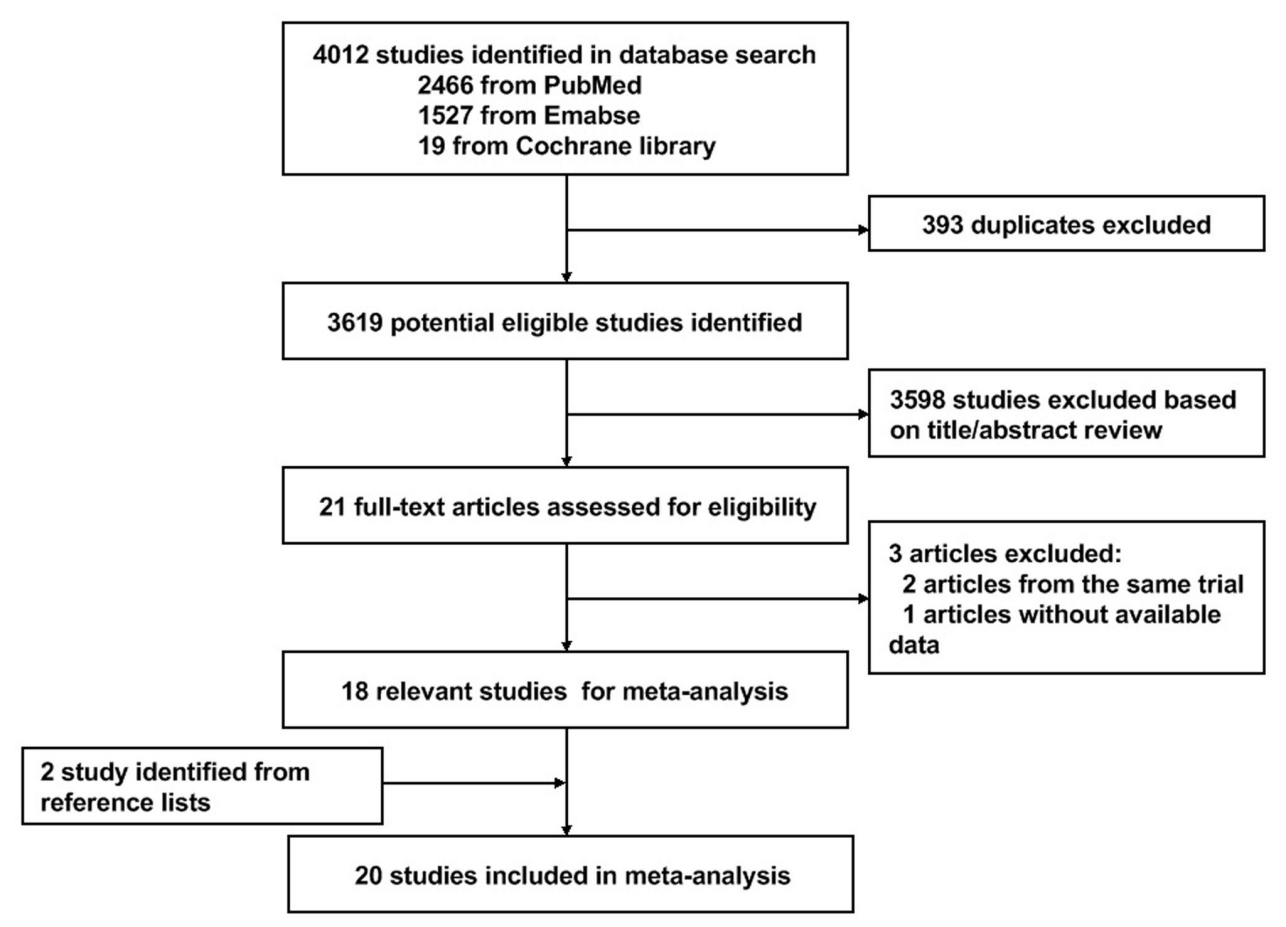
Flow diagram of screened, excluded, and analysed publications.

**Figure 2:**
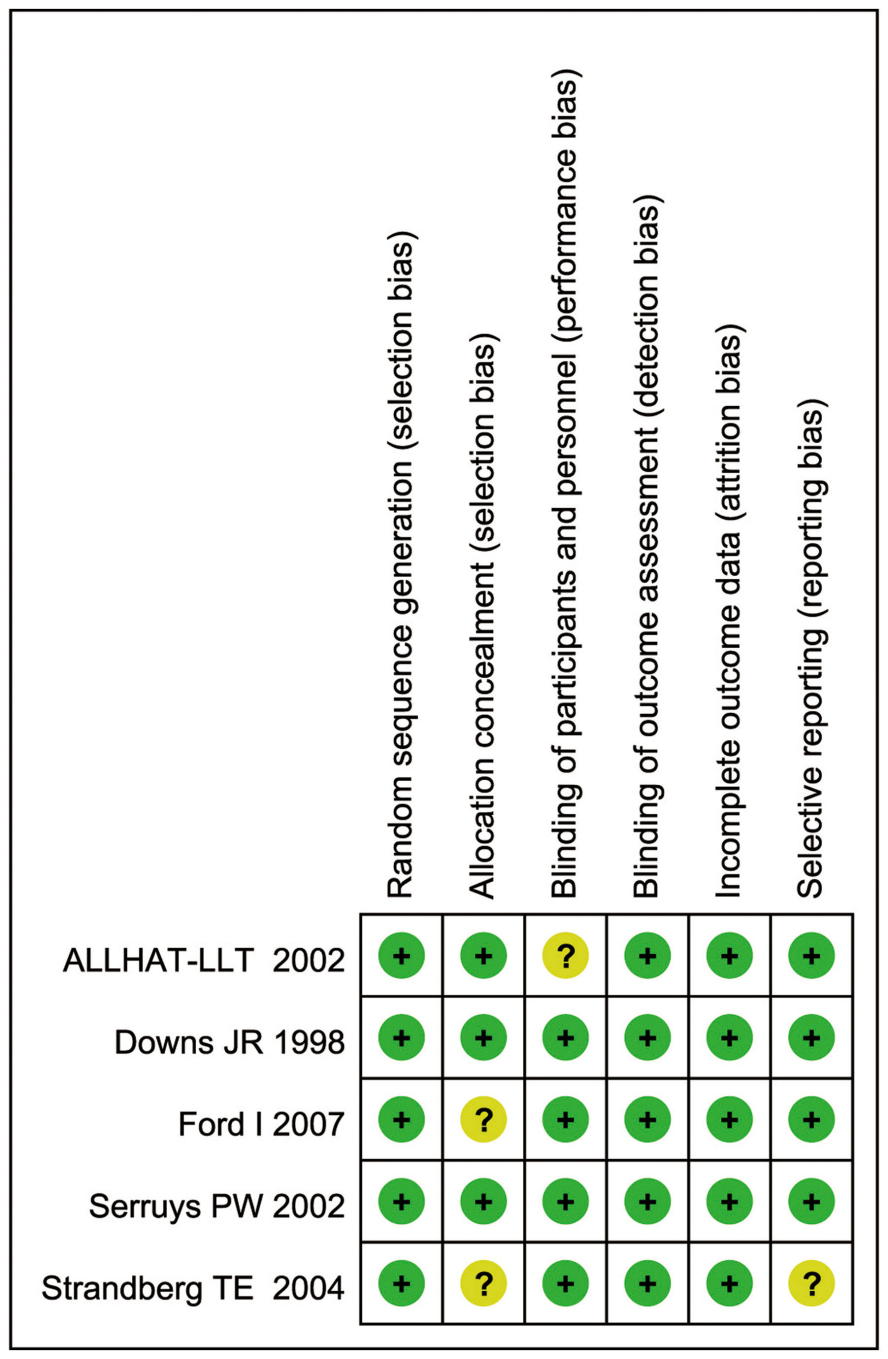
Methodological quality of included randomized controlled trials: review authors’ opinion on each item of bias risk based on Cochrane handbook. “+”, “-” or “?” reflected low risk of bias, high risk of bias and uncertain of bias respectively. ALLHAT-LLT: The Antihypertensive and Lipid-Lowering Treatment to Prevent Heart Attack Trial.

Figure **1** shows the flow diagram for study inclusion. A total of 4012 citations were identified during the initial search. On the basis of the title and abstract, we identified 21 papers. After detailed evaluation, three studies were excluded for reasons described in Figure **1**. Two studies were identified from reference lists. At last, the remaining 20 studies published between 1998 and 2012 were included in the meta-analysis, with five RCTs[30–34], eight cohort studies[35–42], and seven case–control studies[43–49] (Baseline data and other details are shown in Table **1**). A total of 4,980,009 participants, including 37,560 lung cancer cases were involved. Of the 20 included studies, nine studies were conducted in America, nine in Europe, and the remaining two in Asia. Further, six studies[38,41,42,45,48,49] were reported RR estimates of the association between long-term statin use and risk of lung cancer (Table **2**). Figure 2. illustrates our opinion about each item of bias risk for included RCTs, most of the items were at“low risk” based on Cochrane handbook, suggesting a reasonable good quality of RCTs. Table 3 summarizes the quality scores of cohort studies and case-control studies. The Newcastle-Ottawa Scale scores for the included studies ranged from 4 to 9, with a median 6; 9 studies (60%) were deemed to be of a high quality (≥6). 

**Table 1:** Study characteristics.

Author	Year	Country	Study design	Study period	Treated n/N or cases n/N	Contros n/N	Statin type	Confounders for adjustment
Downs JR	1998	USA	RCT	1990-1997	22/3,304	17/3,301	L	Randomization
Blais L	2000	Canada	case-control	1988–1994	NR/70	NR/700	L, P, S	age, sex, use of fibric acid, use of other lipid-reducing agents, previous benign neoplasm, year of cohort entry, the score of comorbidity
Serruys PW	2002	Netherlands	RCT	1996-1998	5/844	3/833	F	Randomization
ALLHAT-LLT	2002	USA	RCT	1994-2002	63/5,170	78/5,185	P	Randomization
Strandberg TE	2004	Nordic countries	RCT	1988-1994	25/2,221	31/2,223	S	Randomization
Graaf MR	2004	Netherlands	case-control	1995–1998	NR/449	986/16,976	A, C, F, P, S	age, sex, geographic region, follow-up time, calendar time, diabetes mellitus, chronic use of diuretics, use of ACE inhibitors,use of calcium antagonists, use of NSAIDs, use of hormones, other lipid-lowering therapies, familiar hypercholesterolemia
Kaye JA	2004	UK	case-control	1990–2002	43/605	1066/14,844	NR	age, BMI,smoking
Friis S	2005	Denmark	cohort	1989–2002	73/12,251	3326/336,011	A, C, F, L, P, S	age, sex, calendar period, use of NSAIDs, use of hormone, use of cardiovascular drugs
Sato S	2006	Japan	cohort	1991-1995	1/179	1/84	P	age, sex, total serum cholesterol level, smoking
Ford I	2007	UK	RCT	1989-1991	102/3,291	109/3,286	P	Randomization
Coogan PF	2007	USA	case-control	1991-2005	31/464	190/3,900	NR	age, sex, BMI, interview year, study center, alcohol consumption, race, years of education, smoking, use of NSAID
Khurana V	2007	USA	case-control	1998-2004	1,994/7,280	161,668/476,453	NR	age, sex, race, BMI, smoking, alcohol use, diabetes mellitus
Setoguchi S	2007	USA	cohort	1994–2003	179/24,439	37/7,284	A, C, F, L, P, S	age, use of NSAIDs, use of hormones, diabetes mellitus, comorbidity score, number of physician visits, prior hospitalization, arthritis, obesity, smoking
Friedman GD	2008	USA	cohort	1994-2003	614/361,859	NR/NR	A, C, F, L, P, R, S	smoking, use of NSAIDs, calendar year
Farwell WR	2008	USA	cohort	1997-2005	436/37,248	431/25,594	A, F, L, P, S	age, weight, thyroid disease, diabetes mellitus, hypertension, cardiovascular disease, renal failure, chest pain, aspirin use, mental illness, alcoholism, lung disease, smoking, total cholesterol
Haukka J	2010	Finland	cohort	1996–2005	112/2,333	135/2,796	A, C, F, L, P, S	sex, age, follow-up period
Hippisley-Cox J	2010	England & Wales	cohort	2002–2008	NR/225,922	NR/1,778,770	A, F, P, R, S	age, sex, comorbidity score, BMI, use of NSAID, smoking, hypertension, use of hormones
Jacobs EJ	2011	USA	cohort	1997-2007	98/47,814 person-years	1,184/707,602 person-years	F, L, P, S	age, sex, race, education, smoking, use of NSAIDs, BMI, physical activity, history of elevated cholesterol, diabetes, heart disease, hypertension
Vinogradova Y	2011	UK	case-control	1998-2008	1,998/10,163	7,621/42,415	A, P, S	diabetes, rheumatoid arthritis, hypertension, BMI, smoking,use of NSAIDs, cyclooxygenase-2 inhibitors and aspirin, hormone replacement therapy, comorbidities, smoking, socioeconomic status
Cheng MH	2012	Taiwan	case-control	2005-2008	61/297	294/1,188	A, F, L, P, R, S	tuberculosis, diabetes, use of NSAIDs, hormone replacement therapy, other lipid-lowering drugs, number of hospitalizations

NR = Not Reported; Treated n/N = No. of cases in the treated group, for cohort studies; cases n/N = No. of exposed in the cases, for case–control studies; Statin type: A= Atorvastatin, C = Cerivastatin, F= Fluvastatin, L = Lovastatin, P= Pravastatin, R= Rosuvastatin, S= Simvastatin; ALLHAT-LLT: The Antihypertensive and Lipid-Lowering Treatment to Prevent Heart Attack Trial

**Table 2:** Studies evaluating the association between long-term statin use and risk of total lung cancer.

Study	year	Study design	RR	95% CI	Definition of "long-term" statin use
Coogan PF	2007	case-control	0.9	0.4-2.1	≥5 years
Khurana V	2007	case-control	0.23	0.2-0.26	>4 years
Setoguchi S	2007	cohort	1.02	0.59-1.74	≥3 years
Friedman GD	2008	cohort	1.06	0.88-1.28	>5 years
Jacobs EJ	2011	cohort	1.08	0.93-1.25	≥5 years
Vinogradova Y	2011	case-control	1.17	0.95-1.45	≥6 years

RR = Relative risk; CI = Confidence interval

**Table 3:** Methodological quality of included cohort studies and case–control studies based on the Newcastle–Ottawa Scale.

Case-control studies	Selection	Comparability	Exposure	Total score
Blais L 2000	3	1	1	5
Graaf MR 2004	2	1	1	4
Kaye JA 2004	4	2	2	8
Coogan PF 2007	2	2	1	5
Khurana V 2007	2	2	1	5
Vinogradova Y 2011	3	2	1	6
Cheng MH 2012	2	1	1	4
Cohort studies	Selection	Comparability	Outcome	Total score
Friis S 2005	3	1	2	6
Sato S 2006	1	1	3	5
Setoguchi S 2007	4	1	2	7
Friedman GD 2008	4	1	1	6
Farwell WR 2008	4	2	3	9
Haukka J 2010	3	1	3	7
Hippisley-Cox J 2010	3	2	3	8
Jacobs EJ 2011	3	2	3	8

### Main analysis

**Figure 3:**
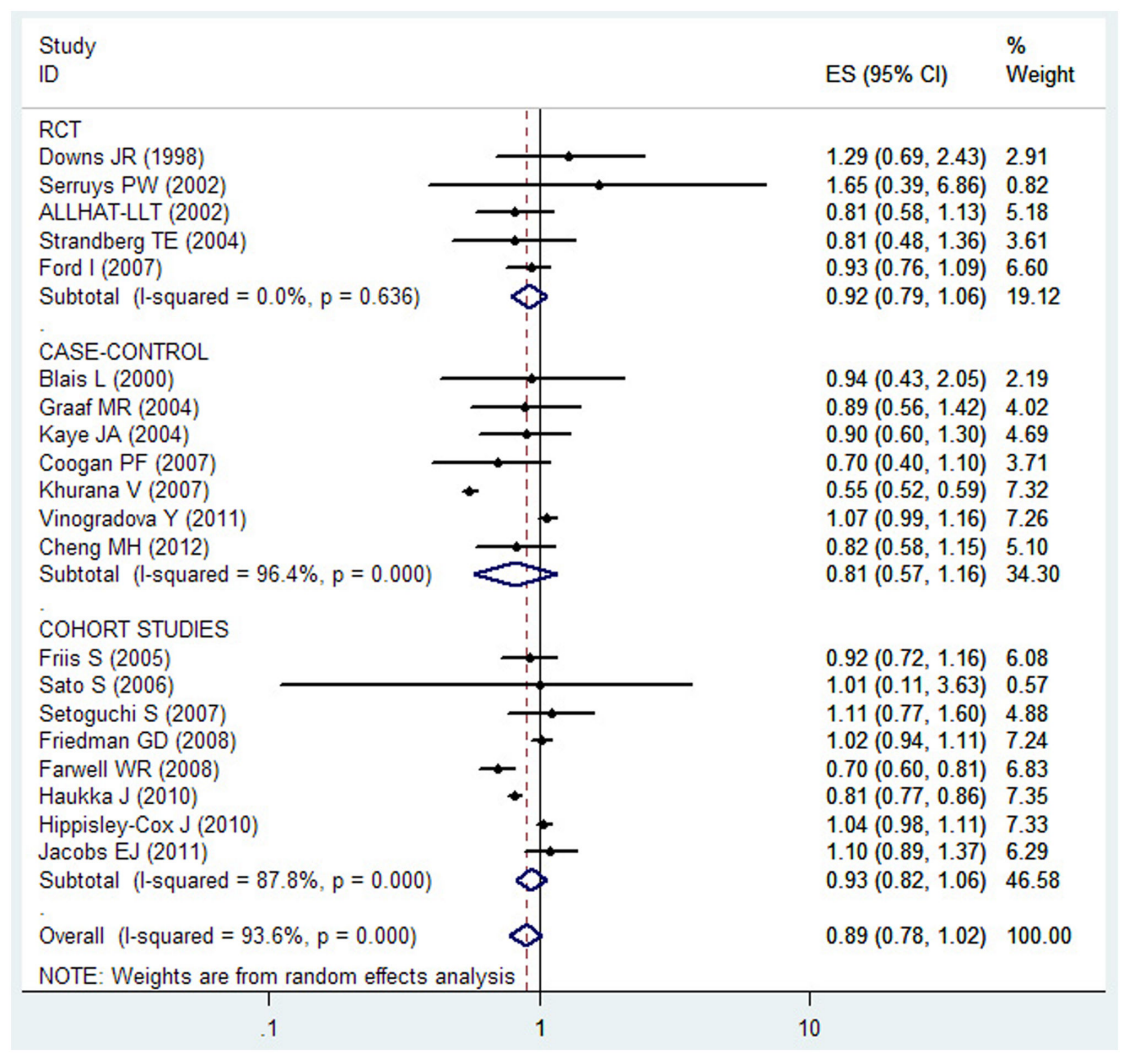
Forest plot: overall meta-analysis of statin use and lung cancer risk. Squares indicated study-specific risk estimates (size of square reflects the study-statistical weight, i.e. inverse of variance); horizontal lines indicate 95% confidence intervals; diamond indicates summary relative risk estimate with its corresponding 95% confidence interval.

**Figure 4:**
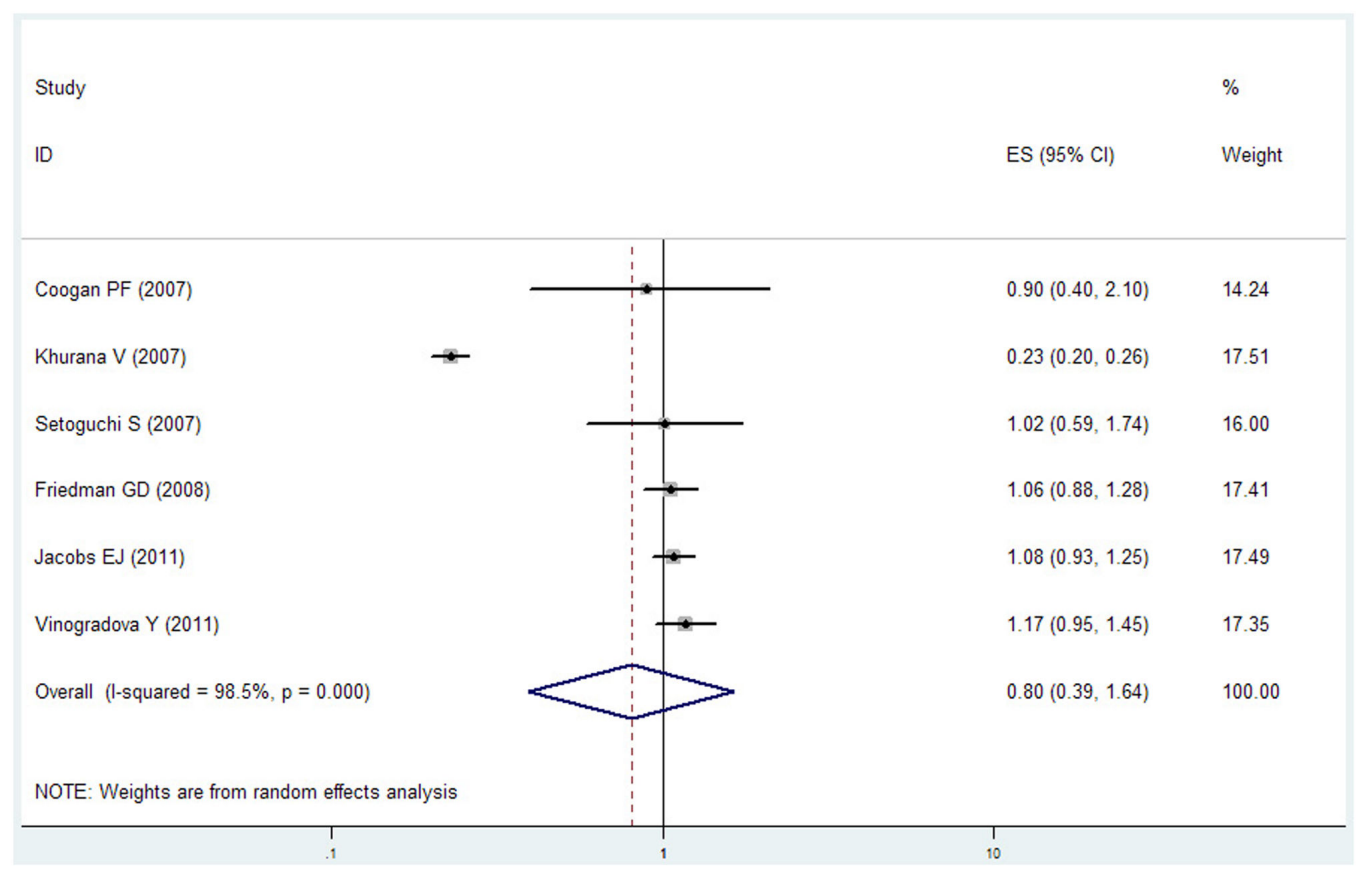
Forest plot: long-term statin use and risk of lung cancer. Squares indicated study-specific risk estimates (size of square reflects the study-statistical weight, i.e. inverse of variance); horizontal lines indicate 95% confidence intervals; diamond indicates summary relative risk estimate with its corresponding 95% confidence interval.

Because of significant heterogeneity (P < 0.001, I^2^ = 93.6%) was observed, a random-effects model was chosen over a fixed-effects model, and we found that statin use did not significantly affect the risk lung cancer (RR = 0.89, 95% CI [0.78, 1.02]). Both multivariable adjusted RR estimates with 95 % CIs of each study and combined RR are shown in Figure **3**. The calculated combined RR for lung cancer in long-term statin use was found to be 0.80 (95% CI [0.39 , 1.64]), presented in Figure **4**.

### Subgroup analyses, sensitivity analysis and cumulative meta-analysis

**Figure 5:**
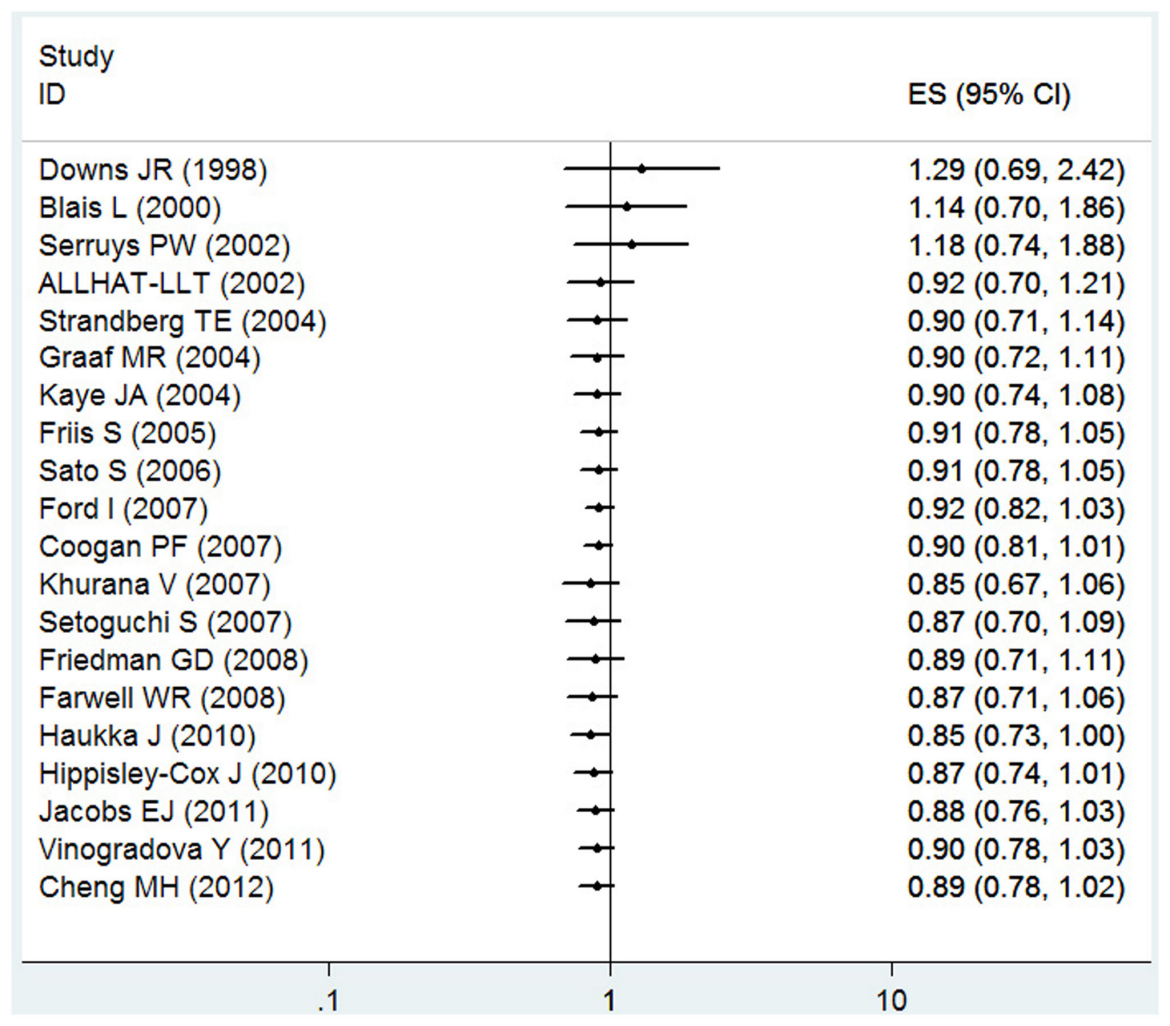
Forest plot: cumulative meta-analysis of statin use and lung cancer risk.

We found no significant association between statin use and risk of lung cancer among RCTs (RR= 0.92, 95%CI [0.79, 1.06]), cohort studies (RR= 0.93, 95%CI [0.82, 1.06]) as well as case–control studies (RR= 0.81, 95% CI [0.57, 1.16]), presented in Table **4**. When stratified the various studies by study location, no significant association was noted among studies conducted in America (RR= 0.84, 95%CI [0.62, 1.13]), Europe (RR= 0.95, 95%CI [0.82, 1.09]), and Asia (RR= 0.83, 95%CI [0.59, 1.16]). When we examined if thorough adjustment of potential confounders could affect the combined RR, it was observed that studies with higher control for potential confounders (n ≥ 8) as well as studies with lower control (n ≤ 7) presented no significant association (RR = 0.96, 95%CI [0.83, 1.09] and RR = 0.82, 95%CI [0.65, 1.04] , respectively)(Table **4**). To test the robustness of association and characterize possible sources of statistical heterogeneity, sensitivity analyses were carried out by excluding studies one-by-one and analyzing the homogeneity and effect size for all of rest studies. Sensitivity analysis indicated that the study by Khurana V et al.[48] contributed most to the variability among all studies. Moreover, no significant variation was observed in combined RR by excluding any of the studies, confirming the stability of present results. A cumulative meta-analysis of total 20 studies was carried out to evaluate the cumulative effect estimate over time. In 1998, DownS JR et al reported an effect estimate of 1.29 (95% CI [0.69, 2.42]). Between 2000 and 2005, seven studies were published, with a cumulative RR being 0.91 (95% CI [0.78, 1.05]). Between 2006 and 2012, 12 more publications were added cumulatively, resulting in an overall effect estimate of 0.89 (95% CI [0.78, 1.02]) (Figure **5**).

**Table 4:** Overall effect estimates for lung cancer and statin use according to study characteristics.

	No. of studies	Pooled estimate		Tests of heterogeneity	
		RR	95% CI	P value	I^2^(%)
All studies	20	0.89	0.78-1.02	<0.001	93.60
Study design					
RCT	5	0.92	0.79-1.06	0.636	0.00
Cohort	8	0.93	0.82-1.06	<0.001	87.80
Case–control	7	0.81	0.57-1.16	<0.001	96.40
Study population					
America	9	0.84	0.62-1.13	<0.001	96.20
Europe	9	0.95	0.82-1.09	<0.001	89.70
Asian	2	0.83	0.59-1.16	0.819	0.00
Adjusted for confounders					
n ≥ 8 confounders	7	0.96	0.83-1.09	<0.001	79.30
n ≤ 7 confounders	8	0.82	0.65-1.04	<0.001	95.50
Results for long-term statin use	6	0.80	0.39-1.64	<0.001	98.50
Adjustment for smoking					
Yes	10	0.89	0.71-1.11	<0.001	96.90
No	5	0.89	0.75-1.06	0.958	0.00

RR = Relative risk; CI = Confidence interval

### Publication bias

**Figure 6:**
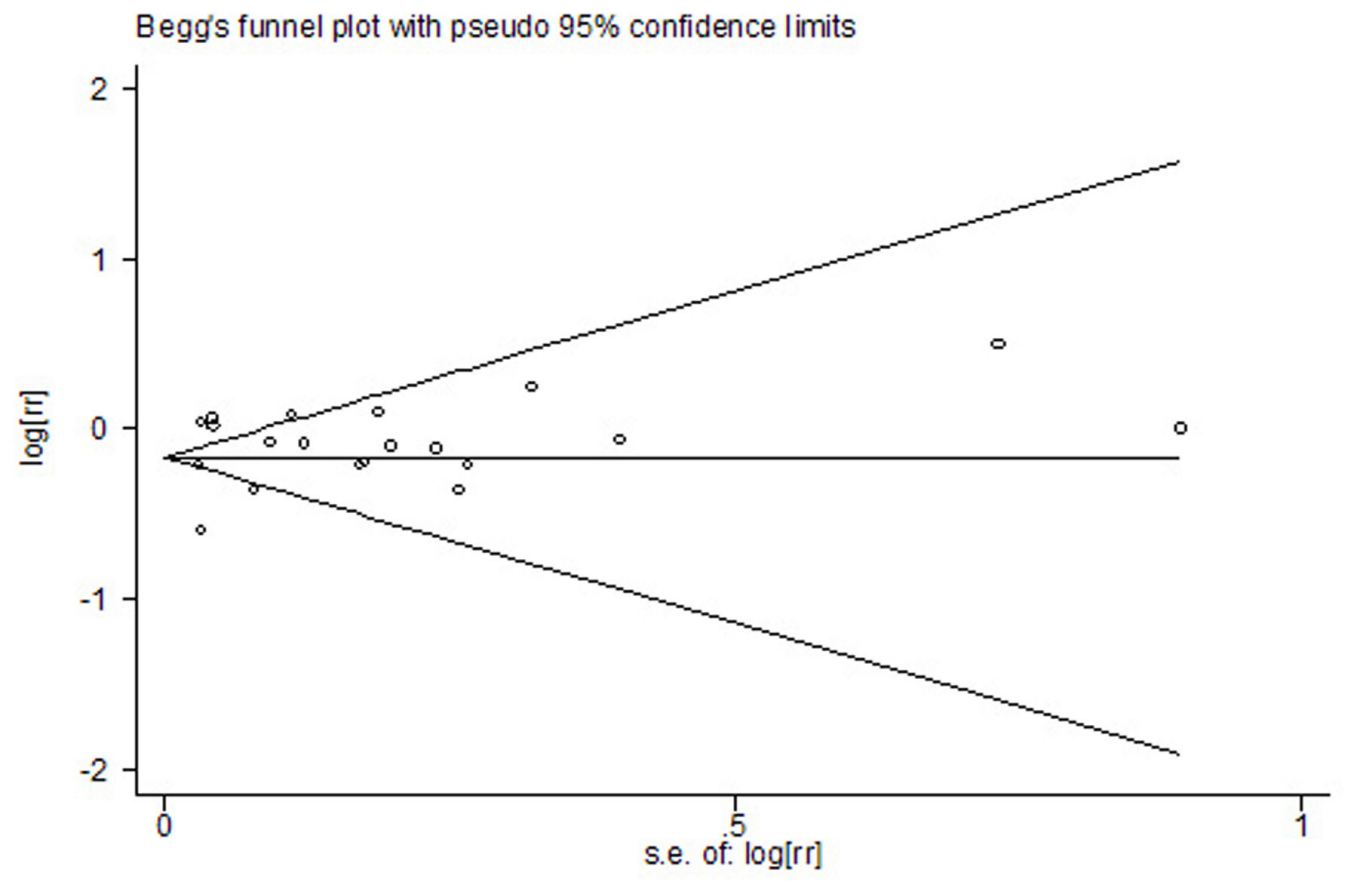
Funnel plot for publication bias in the studies investigating risk for lung cancer associated with use of statins. No publication bias was observed among studies using Begg’s P value ( P = 0.56) and Egger’s ( P = 0.59) test, which suggested there was no evidence of publication bias.

In the present meta-analysis, no publication bias was observed among studies using Begg’s P value (P = 0.56); Egger’s ( P = 0.59) test, which suggested there was no evidence of publication bias (Figure **6**).

## Discussion

In the past decade, the role of statins in the development of cancer has been increasingly understood. The results of meta-analysis conducted by Undela K et al. did not support the hypothesis that statins have a protective effect against breast cancer, however, there was a reduction in risk of breast cancer recurrence in statin users[50]. Consistently, Cui X et al’s meta-analysis suggested that there was no significant association between statin use and pancreatic cancer risk[51]. However, the meta-analysis conducted by Pradelli D et al suggested that statins were inversely related to the risk for liver cancer, with an over 40% decrease in liver cancer risk among statin users, irrespective of the duration of statin exposure[52]. The present meta-analysis included 20 clinical studies currently available (five RCTs, eight cohort studies, and seven case–control studies). Finally, we found no substantial evidence for reduction in lung cancer risk among statin users as compared to non-users, when statins were taken at daily doses for cardiovascular event prevention. In the present meta-analysis, significant heterogeneity was observed among all studies. Therefore, a random-effects model was chosen over a fixed-effects model to determine the pooled RR estimates in our meta-analysis. Sensitivity analysis indicated that the study by Khurana V et al.[48]contributed most to the variability among all studies. The study population in the study of Khurana V et al. consisted solely of veterans with active access to health care and thus they were more likely to be prescribed a statin than the general population. Moreover, an omission of any studies did not significantly alter the magnitude of observed effect, suggesting a stability of our findings. In our subgroup analyses, the results were not substantially affected by study design, study location, and confounder adjustment. RCTs, cohort and case–control studies alone showed no significant association between statin use and risk of lung cancer. Cumulative meta-analysis did not show a significant change in trend of reporting risk of lung cancer in statin users between 1998 and 2012. Furthermore, our results demonstrated that long-term statin use did not significantly reduce the risk of lung cancer incidence. However, we should treat this result with caution. Firstly, patterns of statin use were different in the included studies. In many cases, drug use was irregular, with months of non-use between periods of use. Therefore, cumulative amount of statin defined daily doses (DDDs) could be small despite its long duration. Secondly, the definition of ‘‘long-term use’’ was different among the included studies. Thirdly, only six studies were reported RR estimates of the association between long-term statin use and risk of lung cancer. 

Despite some experimental studies have found that statin may induce apoptosis[14–18], inhibit tumor growth[19–22], angiogenesis[23], as well as metastasis[24], our results suggested there was no conclusive preventive effect of statin use on lung cancer risk. These findings were in line with the recent meta-analysis of statin use and overall cancer risk[53]. We should notice that the inhibitory effect of statins on lung cancer cells has thus far been tested only in vitro and may behave differently in vivo. As we know, statins are selectively localized to the liver, and less than 5% of a given dose reaches the systemic circulation. Thereby, the usefulness of statins as chemopreventive agents for lung cancer is doubted given their selective hepatic uptake and low systemic availability[54]. Previous meta-analyses suggested that there was no significant association between statin use and breast and pancreatic cancer risk[50,51], however, statin had a protective effect against liver cancer[52], which supports the opinion above. Further, statins have been shown to increase regulatory T-cell numbers and functionality in vivo[55–57]; both lipophilic and hydrophilic statins decrease natural killer cell cytotoxicity[58]. These immunosuppressive effects of statins might impair host antitumor immune responses, suggesting an opposing effect on tumor development, which should be considered. In one of the included studies[46], Graaf et al presented the effect of duration of statin use and dose. However, neither dose - response nor duration - response relationship was found. The absence of a significant dose-response or duration - response weighs against a causal inference. 

The study by Khurana et al[48] found that statin use ≥6 month was associated with a statistically significant risk reduction of lung cancer by 55%(OR = 0.45, 95%CI = 0.42–0.48). We noted that the study population in the study of Khurana et al. consisted solely of veterans with active access to health care and thus they were more likely to be prescribed a statin than the general population. Further, 97.9% of the participants in their study were men. Cheng MH et al[44] investigated the association between statin use and lung cancer risk in female population individually, and they found that statin use was not associated with the risk of female lung cancer. Another study by Hippisley-Cox et al[40] investigated statin use and lung cancer risk among male or female population independently, and the result revealed that statin use was not associated with lung cancer risk in both female(OR =1.00, 95%CI =0.81-1.23) and male population(OR =1.05, 95%CI =0.97-1.13). Therefore, it’s not clear whether statin use was associated with lung cancer risk among male or female population, especially male population. This topic need further discussion in the future when there are enough studies investigating statin use and lung cancer risk among male or female population independently.

The strength of the present analysis lies in inclusion of 20 studies(five RCTs, eight cohort studies, and seven case–control studies). Publication bias, which, due to the tendency of not publishing small studies with null results, was not found in our meta-analysis. Furthermore, our findings were stable and robust in the subgroup analyses and sensitivity analyses.

Our meta-analysis has several limitations. First, we did not search for unpublished studies, so only published studies were included in our meta-analysis. Therefore, publication bias may have occurred although no publication bias was indicated from both visualization of the funnel plot and Egger’s test. Second, we haven’t done subgroup meta-analyses of different gender or lung cancer histology, for a lack of original data. Finally, the included studies were different in terms of study design and definitions of drug exposure.

In conclusion, the findings of this meta-analysis, suggested that there was no significant association between statin use and risk of lung cancer. More studies, especially RCTs and high quality cohort studies with larger sample size, well controlled confounding factors and longer duration of follow-up are needed to confirm this association in the future.

## Supporting Information

Checklist S1 **PRISMA Checklist.**
(DOC)
